# Targeted Antiepidermal Growth Factor Receptor (Cetuximab) Immunoliposomes Enhance Cellular Uptake *In Vitro* and Exhibit Increased Accumulation in an Intracranial Model of Glioblastoma Multiforme

**DOI:** 10.1155/2013/209205

**Published:** 2013-09-23

**Authors:** Joachim Høg Mortensen, Maria Jeppesen, Linda Pilgaard, Ralf Agger, Meg Duroux, Vladimir Zachar, Torben Moos

**Affiliations:** ^1^Laboratory of Cancer Biology, Biomedicine, Institute of Medicine and Health Technology, Fredrik Bajers Vej 3B, 1.216, Aalborg University, 9220 Aalborg East, Denmark; ^2^Laboratory of Neurobiology, Biomedicine, Institute of Medicine and Health Technology, Fredrik Bajers Vej 3B, 1.216, Aalborg University, 9220 Aalborg East, Denmark; ^3^Laboratory of Immunology, Biomedicine, Institute of Medicine and Health Technology, Fredrik Bajers Vej 3B, 1.216, Aalborg University, 9220 Aalborg East, Denmark; ^4^Laboratory of Stem Cell Research, Biomedicine, Institute of Medicine and Health Technology, Fredrik Bajers Vej 3B, 1.216, Aalborg University, 9220 Aalborg East, Denmark; ^5^Section of Neurobiology, Biomedicine, Institute of Medicine and Health Technology, Fredrik Bajers Vej 3B, 1.216, Aalborg University, 9220 Aalborg East, Denmark

## Abstract

Therapeutic advances do not circumvent the devastating fact that the survival rate in glioblastoma multiforme (GBM) is less than 5%. Nanoparticles consisting of liposome-based therapeutics are provided against a variety of cancer types including GBM, but available liposomal formulations are provided without targeting moieties, which increases the dosing demands to reach therapeutic concentrations with risks of side effects. We prepared PEGylated immunoliposomes (ILs) conjugated with anti-human epidermal growth factor receptor (EGFR) antibodies Cetuximab (**α**-hEGFR-ILs). The affinity of the **α**-hEGFR-ILs for the EGF receptor was evaluated *in vitro* using U87 mg and U251 mg cells and *in vivo* using an intracranial U87 mg xenograft model. The xenograft model was additionally analyzed with respect to permeability to endogenous albumin, tumor size, and vascularization. The *in vitro* studies revealed significantly higher binding of **α**-hEGFR-ILs when compared with liposomes conjugated with isotypic nonimmune immunoglobulin. The uptake and internalization of the **α**-hEGFR-ILs by U87 mg cells were further confirmed by 3D deconvolution analyses. *In vivo*, the **α**-hEGFR-ILs accumulated to a higher extent inside the tumor when compared to nonimmune liposomes. The data show that **α**-hEGFR-ILs significantly enhance the uptake and accumulation of liposomes in this experimental model of GBM suggestive of improved specific nanoparticle-based delivery.

## 1. Introduction

Gliomas account for almost all primary tumors in the central nervous system (CNS) among which glioblastoma multiforme (GBM) is the most malignant and invasive. In spite of some therapeutic improvements from neurosurgery, radiation therapy, and pharmacology, the 5-year median survival rate is less than 5%, which clearly justifies attempts to improve treatment. The epidermal growth factor receptor (EFGR) is a transmembrane glycoprotein with its intracellular domain acting as a tyrosine kinase and its extracellular part acting as a receptor with high affinity for EGFR [1]. EGFR is highly expressed in cancer cells in more than 40% of GBM cases, and the mutated form of EGFR, EGFRvIII, is additionally expressed in more than 40% of GBM cases expressing EGFR [2, 3], clearly indicating that EGFR could play a role in GBM pathogenesis. Furthermore, as EGFR and EGFRvIII are substantially expressed by the cancer cells in GBM, these receptors are amendable for targeted therapy [4].

Liposome-based therapeutics are usable for treatment of a variety of cancer types, but current available liposomes for human use are not provided conjugated with targeting molecules, which increases the demand for dosage to reach a therapeutic acceptable concentration near the cancer cells and also increases the risks of side effects [5]. Accordingly, target-based therapeutics consisting of protein ligands or antibodies conjugated to liposomes are widely investigated for drug delivery to cancer cells specifically expressing certain proteins adaptable for targeting. Targeted therapy using systemic injection of antibodies conjugated to chemotherapeutic-loaded liposomes, immunoliposomes, causes significant tumor cytotoxicity and growth inhibition compared to nontargeted liposomes [6–8], hence making immunoliposomes a promising tool for future treatment in oncology. Upon binding to the EGFR, receptor-mediated endocytosis and transport to the cytosol of EGF occurs suggesting that the EGFR denotes an excellent target for drug delivery to EGFR overexpressing cancer cells in GBM [9].

To enable immunoliposome therapy to cancer cells in GBM, the immunoliposomes must pass the fenestrated tumor endothelial cells formed by capillaries of the host organism growing towards the tumor, a process called tumor angiogenesis. The size of fenestrations of the tumor endothelial cells allow nanoparticles with size diameters of 100–550 nm in intracranial tumor xenografts and up to 380–2000 nm in subcutaneous tumor xenografts to pass from the circulation into the tumor interstitium where they can access the cancer cells. Furthermore, molecules may also become trapped in this interstitium a phenomenon generally referred to as the enhanced penetration and retention (EPR) effect [10, 11]. The EPR effect will increase the likelihood of trapping molecules inside the tumor where the endothelium is leaky. However, in case of the brain the EPR effect is generally lower than in many other tissues due to molecules released from the vicinity of the ingrowing capillaries and direct contact of nonneuronal cells forming the neurovascular unit surrounding the endothelial cells, for example, astrocytes, pericytes, and perivascular macrophages [12–14]. In the present study, we demonstrate that conjugation with a commercial available monoclonal anti-human EGFR antibody, Cetuximab, significantly enhances the uptake and accumulation of liposomes in a xenograft animal model of GBM.

## 2. Materials and Methods

### 2.1. Materials

The reagents were obtained from the following sources: Dulbeccos Modified Eagles Medium (DMEM) (Lonza, Cat. No. BE12-614F), LabTek permanox chamber slides (Nunc, Cat No. 177445), fetal calf serum (FCS) (Invitrogen, Cat. No. 10099-141), fluorescence mounting medium (DAKO, Cat. No. S3023), normal goat serum (DAKO, Cat. No. X0907), PD-10 desalting column (GE-Healthcare, Cat. No. 17-9323-14), penicillin/streptomycin (Invitrogen, Cat. No. 15070-063), polycarbonate membranes for manual extrusion (Nucleopore Track-Etch Membrane Filtration Products, Whatman, Avanti Polar Lipids), had a pore sizes of 0.2 *μ*m (Cat. No. 800281), 0.1 *μ*m (Cat. No. 800306), and 0.05 *μ*m (Cat. No. 800308), succinimidyl acetylthioacetate (SATA) (Pierce, Cat. No. 26102), TissueTek (Sakura, Cat. No. 4583 O.C.T), 50 kDa Vivaspin 6 ultrafiltration device (GE-Healthcare, Cat. No. 28-9323-18). The following reagents were from Avanti Polar Lipids: Cholesterol (Cat. No. 700000P), 1,2-dipalmitoyl-sn-glycero-3-phosphoethanolamine-N-[methoxy-(polyethylene glycol)-2000] (mPEG2000-PE) (Cat. No. 880160P), 2-distearoyl-sn-glycero-3-phosphoethanolamine-N-[maleimide (polyethylene glycol)-2000] (Cat. No. 880126P), soy phosphatidylcholine (Cat. No. 840054P). The following reagents were form Sigma-Aldrich: Bovine serum albumin (BSA) (Cat. No. A9647), 4′,6-diamidino-2-phenylindole (DAPI) (Cat. No. D9542), 3,3′-dioctadecyloxacarbocyanine perchlorate (DiO) (Cat. No. D4292), N-N-dimethylformamide (Cat. No. D4551), nonimmune IgG from human serum (Cat. No. I4506), 99.9% hydroxylamine (Cat. No. 55459), 4% paraformaldehyde (Cat. No. P6148), 4B sepharose (Cat. No. 43200), Tween 20 (Cat. No. P9416). Antibodies and vendors: Alexa Fluor 488 goat-anti-human (H+L) (Molecular Probes, Invitrogen, Cat. No. A-11013), Alexa Fluor 555 goat-anti-human (H+L) (Molecular Probes, Invitrogen, Cat. No. A-21433), Alexa Fluor 555 donkey-anti-goat (Molecular Probes, Invitrogen, Cat. No. A-21432), Alexa Fluor 488 goat-anti-rabbit (Molecular Probes, Invitrogen, Cat. No. A-31565), Alexa Fluor 555 goat-anti-rabbit (Molecular Probes, Invitrogen, Cat. No. A-21427), monoclonal anti-human epidermal growth factor receptor antibody (Merckserono, Erbitux), polyclonal rabbit anti-laminin antibody (DAKO, Cat. No. Z0097), and polyclonal goat-anti-mouse albumin (Nordic Biosite, Cat. No. A90-134A).

### 2.2. Cell Lines

The cell lines used in the study were U87 mg (American Type Culture Collection [ATCC], Cat. No. HTB-14) and U251 mg (Health Protection Agency Culture Collection [HPA Culture Collection], Cat. No. 09063001). The cell lines U87 mg and U251 mg were cultured in DMEM supplemented with 10% (FCS) and 1% penicillin/streptomycin. Cell cultures were kept in a humidified atmosphere containing 5% CO_2_ buffered with ambient air at 37°C. The cell medium was changed twice a week.

### 2.3. Liposome Preparation

Liposomes were prepared from soy phosphatidylcholine (soyPC), cholesterol, 2-distearoyl-sn-glycero-3-phosphoethanolamine-N-[maleimide (polyethylene glycol)-2000] (DSPE-PEG2000-Mal), 1,2-dipalmitoyl-sn-glycero-3-phosphoethanolamine-N-[methoxy-(polyethylene glycol)-2000] (mPEG2000-PE), and the fluorescent probe DiO in a molar ratio of 65 : 30 : 2 : 3 : 0.5. The lipids used for this procedure were all dissolved in chloroform and transferred to a round-bottom flask. A thin lipid film was formed by evaporating the chloroform with a stream of gaseous nitrogen for 30 minutes at room temperature. The resulting lipid film was hydrated in HEPES Buffer (10 mM HEPES, 136 mM NaCl, and 1 mM EDTA). To ensure that the lipid film was completely dissolved, the flask was immediately vortexed, and to allow complete hydration the flask was incubated at room temperature on a shaker for one hour. The homogenous liposomes were prepared by a manual extrusion technique by passing through polycarbonate membranes 20 times for each filter with pore sizes of 0.2 *μ*m, 0.1 *μ*m, and finally 0.05 *μ*m.

### 2.4. Formation of Immunoliposomes

The anti-human-EGFR antibody (Erbitux) was used to form immunoliposomes (*α*-hEGFR-IL's). Control liposomes were either prepared by conjugation with nonimmune IgG from human serum (hHIgG-IL's) or left unconjugated (naked liposomes). In order to ensure that the amine group of free amino acids theoretically present in the antibody solutions did not interfere with the conjugation process, they were removed by a buffer exchange with HEPES buffer using gel filtration chromatography, and PD-10 desalting column was prepared according to the manufacture protocol (GE Healthcare, UK). SATA was dissolved in N-N-dimethyl formamide in a ratio of 1 : 100. The SATA was employed for thiolation, which is necessary for antibodies to crosslink with the maleimide group of the DSPE-PEG2000-maleimide lipid. The SATA solution was mixed with the antibody solution in a molar ratio of 8 : 1 SATA : antibody and incubated for 45 minutes at room temperature during continuous rotation. Unbound SATA was removed, according to the manufacture protocol by using a 50 kDa Vivaspin 6 ultrafiltration device (GE-Healthcare, 28-9323-18). The protein concentration of the antibodies was determined by UV spectroscopy (Implen NanoPhotometer).

In order for the SATA to crosslink with the maleimide groups, the sulfhydryl groups were deacetylated by mixing the SATA/antibody solution with hydroxylamine solution (0.5 M hydroxylamine HCl; 0.5 HEPES M HEPES; 25 mM EDTA) and incubated for one hour at room temperature before mixing the SATA : antibody solution with liposomes. Finally, the conjugation was performed by mixing the liposomes with the deacetylated SATA : antibody solution in a molar ratio of ratio of 1 : 1000 for DSPE-PEG2000-maleimide:antibody and incubated for 2 hours at room temperature followed by incubation on a rotator at 4°C overnight. Unbound antibody and self-aggregated liposomes were separated from immunoliposomes by gel filtration chromatography using a 4B sepharose gel.

Mean particle size of the various liposomes was determined by dynamic light scattering and the zeta potential by laser Doppler electrophoresis using a Zetasizer Nano ZS (Malvern). Determination of particle size using the Zetasizer Nano ZS generates a Z-average value of mean particle size, polydispersity of the size distribution, and the mean size of individual peaks present in the particle suspension. All measurements were performed on four separate samples and data was analyzed using Malvern Zetasizer Software v.6.2.

The concentrations of the conjugated antibodies were determined using the RC DC Protein Assay (BioRad, Cat. No. 500-0121). A standard curve was prepared consisting of five dilutions ranging from 0.2 mg/mL to 1.5 mg/mL nonimmune IgG from human serum in HEPES buffer. Liposome samples were diluted 1 : 2 in HEPES buffer to ensure that the samples were within range of the standard curve. All standards and samples were prepared in duplicate. Absorbance was read at 750 nm using a spectrophotometer (Thermo Fischer Scientific, Genesys 10 UV-Vis Scanner) using disposable semimicropolystyrene cuvettes (Sarstedt, Germany). The antibody concentration of the various liposome samples was calculated from the plotted standard curve.

The phosphatidylcholine concentration of the final liposome suspensions was determined using a phosphatidylcholine assay kit (Biovision, Cat. No. K576-100). The assay was performed according to manufacturer's instructions for the colorimetric assay. Absorbance was read at 570 nm using a spectrophotometer (NanoPhotometer, Implen) equipped with a quartz ultramicrocell (Hellma, VWR, Denmark). All readings were corrected for nonspecific background by subtracting the zero value of phosphatidylcholine. The phosphatidylcholine concentration of each sample was calculated from the plotted standard curve, and from these values the total lipid concentration was estimated assuming that phosphatidylcholine comprises approximately 65 mol% of the final liposome-concentration measured in molar amounts. The calculated protein concentration was then correlated to the lipid concentration with calculate the amount of antibodies/nmol liposome.

### 2.5. *In Vitro* Cellular Binding and Internalization of Liposomes

The cellular binding of liposomes to U87 mg and U251 mg cells was investigated *in vitro* to determine the uptake of targeted anti-EGFR liposomes compared to those of unconjugated and nonimmune-IgG conjugated liposomes. The two cell lines both express high levels of EGFR. However, U87 mg was chosen for the *in vivo *studies because a successful U87 mg intracranial xenograft model had already been established in our laboratory. The cellular uptake of green fluorescent liposomes was visualized by fluorescence microscopy, and their targeting potential was quantified by flow cytometric analysis. U87 mg cells were seeded in separate 8 wells LabTek permanox chamber slides 24 hours before initiating the uptake experiments. The liposomes were added to the wells at a concentration of 75 nM (0.0075 mol/L) per 10^5^ seeded cells and incubated for 2 hours at 37°C in cell medium supplemented with 10% (FCS) and 1% penicillin/streptomycin. Unbound liposomes were removed by washing 3 times with 0.1 M PBS (pH 7.0). The cells were fixed in 4% paraformaldehyde for 15 minutes and nuclei stained with DAPI. In order to confirm that the primary anti-human-EGFR antibodies and nonimmune IgG were indeed conjugated to the liposomes, Alexa Fluor 488 goat-anti-mouse secondary antibody was incubated for 45 minutes with the cells after removal of unbound liposomes. Fluorescence images were obtained with an AxioCam MRm (Carl Zeiss International) attached to a Zeiss Axio Observer.Z1 microscope (Carl Zeiss International) using the AxioVision rel. 4.7 software (Carl Zeiss International). For each cell line, a representative Z-stack of 25 stacks was obtained at 400x magnification. In order to eliminate light of different planes from the Z-stack, 3D deconvolution was carried out using AxioVision rel. 4.7 software (Carl Zeiss International). 3D deconvolution was performed using a theoretical point spread function with 25 iterations.

Flow cytometry was used to quantify the targeting potential of the liposomes. Identical liposome concentrations and incubation times were applied during this experiment (75 nmoles/10^5^ cells). However, immediately after the 2-hour incubation, the cells were trypsinized and transferred to an eppendorf tube. Unbound liposomes were removed by washing 3 times in PBS and centrifuged. The liposome targeting potential was evaluated by FlowJo v. 7.6. software. A total of 100,000 cells were analyzed for each cell line, and the experiments were repeated twice. The mean fluorescence intensity (MFI) of the DiO labeled liposomes was expressed in arbitrary units.

### 2.6. Intracranial Tumor Xenograft Model

Male NMRI CD1 nude mice aged 6–10 months were used for inoculation of U87 mg cells. To avoid contamination and infection, all mice were housed in a temperature and humidity controlled ventilated filter cabinet. The animals had free access to food and water during the experiments. The procedures dealing with the handling of animals described in this study were approved by the Danish Experimental Animal Inspectorate under the Ministry of Justice.

The NMRI mice (*n* = 7) were inoculated with U87 mg (10.000 cells/*μ*L) in the striatum. A total volume of 5 *μ*L was inoculated in the striatum of the mice using a Hamilton syringe. The mice were anesthetized by subcutaneous injection of 0.1 mL/10 g body weight of Hypnorm, Dormicum and sterile water in a ratio of 1 : 1 : 1. For the inoculation procedure, the mice were placed in a stereotactic apparatus (Stoelting Lab). The skin was incised at the midline and retracted and the exposed calvarium was disinfected with 1% hydrogen peroxide. The scalp was dried by dabbing with a tissue. A small hole was drilled by a hand-held drill through the skull 1.1 mm lateral from the midline, 1.1 mm dorsal to the bregma, and cells were injected 3.5 mm deep from the brain's surface to implant the striatum according to an atlas of the adult mouse brain [15]. The U87 mg cells were slowly injected to prevent a rapid change in the intracranial pressure, and the syringe was left in for 5 min before retracting the syringe to avoid cells from ascending through the injection canal. Judged from preliminary studies, the total span of the experiments was set to 21 days to ensure sufficient tumor development. However, in case the mice developed signs of considerable tumor burden, defined as loss of more than 20% of the mice initial body weight and neurological signs, for example, balance and gait difficulties, they were immediately euthanized. After 21 days, the mice were injected in the tail vein with 1.0 *μ*mol of liposomes dispersed in 0.2 M HEPES-buffer. The mice were euthanized with Hypnorm-Dormicum and sacrificed by transcardial perfusion fixation with 4% paraformaldehyde in 0.1 M potassium phosphate-buffered saline (KPBS). The brain was then removed and immersed into the fixative at 4°C for 24 hours, after which it was washed for 3 times in KPBS and immersed in 30% sucrose solution in KPBS for a minimum of 48 hours.

### 2.7. Biodistribution of the Liposomes

The mouse brains were embedded in TissueTek (Sakura, Finetek Europe B.V., Netherlands) and sectioned at 40 *μ*m using a cryostat (Microm, Germany). Unstained sections were analyzed to examine the distribution of the fluorescent liposomes. Furthermore, the sections were counterstained with DAPI and antilaminin using immunohistochemistry (see below). 

### 2.8. Immunocyto- and Histochemistry

U87 mg and U251 mg were seeded into eight well LabTek permanox chambers. When cells had reached a confluence level of 70–80%, the medium was removed, and cells were washed 3 times with phosphate buffered saline (PBS). Finally, the cells were fixed with 4% paraformaldehyde by incubation for 15 minutes at room temperature. Prior to any immunocytochemical staining, the cells were incubated with blocking buffer consisting of KPBS, 5% goat, and 2% bovine serum albumin (Sigma-Aldrich) for 1 hour to block unspecific binding. The monoclonal chimeric human/mouse-anti-human EGFR antibody was added at a concentration of 50 *μ*g/mL in incubation buffer (3% normal goat serum, 2% BSA, and 0.3 Tween 20 in PBS) and incubated at 4°C overnight on a belly dancer. Next day, Alexa Fluor 488 goat-anti-human (H+L) was used as a secondary antibody in a 1 : 200 dilution to visualize EGFR-expression. Excess of secondary antibody was removed by washing 3 times with PBS. The cells were then stained with DAPI for 10 minutes in a 1 : 500 dilution and washed 3 times with PBS. Finally, fluorescence mounting medium was applied as antifade reagent. Fluorescence images were obtained with an AxioCam MRm (Carl Zeiss International) attached to a Zeiss Axio Observer.Z1 microscope (Carl Zeiss International) using the AxioVision rel. 4.7 software (Carl Zeiss International).

Immunohistochemical staining was performed on sections from the brain. The sections were washed for 3 times in PBS prior to the staining. The antibodies used were rabbit anti-laminin for capillary staining and human anti-human-EGFR for detecting EGFR-positive cancer cells and goat anti-mouse albumin for identifying endogenous mouse albumin. All sections were left overnight with primary antibodies at 4°C. In some cases, the Alexa Fluor 488 goat anti-human antibody was applied to the sections to enhance the green fluorescence emitted by the liposomes. Goat anti-rabbit Alexa Fluor 488 or 555 was used to stain for laminin, and Alexa Fluor donkey anti-goat was used to visualize endogenous mouse albumin. Secondary antibodies were incubated for two hours at room temperature following counterstaining with DAPI. Fluorescence images were obtained with an AxioCam MRm (Carl Zeiss International) attached to a Zeiss Axio Observer.Z1 microscope (Carl Zeiss International) using the AxioVision rel. 4.7 software (Carl Zeiss International). All images of the brain were taken at the tumor periphery, since tumor vascularization was very low in the centre of the tumor.

### 2.9. Statistical Analysis

Statistical significances between groups in the *in vitro* cellular binding assay were calculated using unpaired Student's *t*-test. Significance was assumed at a *P* value <0.05.

## 3. Results

### 3.1. *In Vitro* and *In Vivo* Expression of Epidermal Growth Factor Receptor in Cell Lines

**Figure 1:**
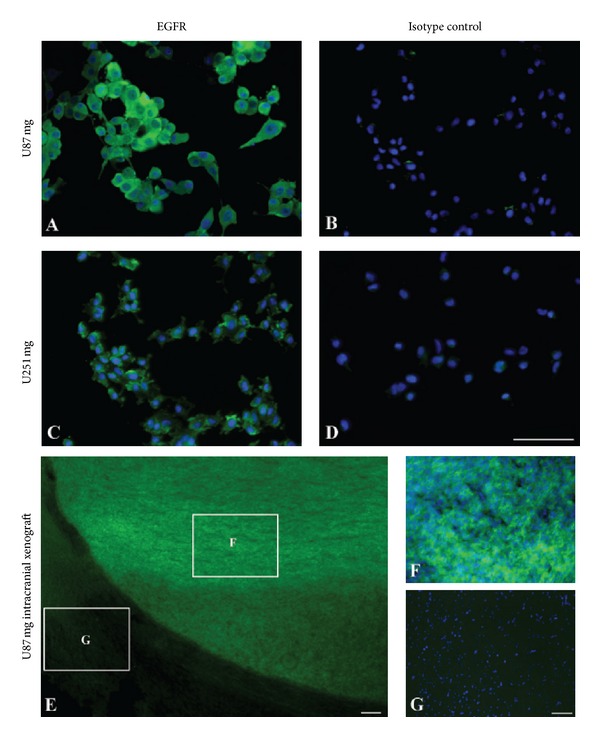
Representative micrographs showing expression of epidermal growth factor receptor (EGFR) *in vitro* and *in vivo*. (A), (C) *In vitro* expression of EGFR in U87 mg (A) and U251 mg (C) cell lines using fluorescent antibodies. The cells are labeled using an anti-human EGFR primary antibody (green) and cellular nuclei demonstrated with 4′,6-diamidino-2-phenylindole (DAPI) (blue) Application of nonimmune isotypic immunoglobulins reveals no labeling of U87 mg (B) and U251 mg (D) cells. (E)–(G) *In vivo* expression of EGFR in the U87 mg intracranial xenograft model. (E), EGFR expression detected in U87 mg cells using a fluorescent secondary antibody (green) shown at low-power magnification. The U87 mg cells form a prominent tumor with a clear demarcation that leaves the surrounding normal brain tissue unlabeled. When examined at larger magnification (cropped areas in (E)), it is evident that EGF-receptors are expressed by U87 mg cells forming the xenograft (F), and not in cells of the normal brain (G). Scale bars = 50 *μ*m ((A), (B), (C), (D), (F), (G)), 100 *μ*m (E).

The expression of EGFR in the U87 mg and U251 mg cell lines appeared very homogeneous with no detectable differences between the two cell lines. Hence, both cell lines revealed extensive EGFR labeling of the cytoplasm and cellular surfaces without labeling of the nucleus (Figures 1(A) and 1(C)). Substitution of the primary antibody with isotopic nonimmune IgG revealed no immunoreactivity within the cells (Figures 1(B) and 1(D)). Likewise, no immunoreactivity was observed when the primary antibody was omitted from the immunoreactions (not shown). When examined in the intracranial xenograft, it was evident that EGFR positive cells were detected in the cells forming a tumor, which contrasted that of neurons and glia of the normal brain tissue (Figures 1(E)–1(G)). When examined at high magnification, the EGFR-immunoreactive cells exhibited a morphology that corresponded to that of U87 mg expressing EGFR *in vitro*. In contrast, neurons and glia of the normal brain tissue were devoid of EGFR-immunoreactivity (Figure 1(G)).

### 3.2. Liposome Characterization

Fluorescence labeled liposomes were prepared with anti-EGFR antibodies or isotypic human immunoglobulins coupled with the DSPE-PEG2000-Mal linker. *α*-hEGFR-ILs were compared to liposomes conjugated with nonimmune human immunoglobulins and naked liposomes with no antibody conjugation with respect to particle size, polydispersity, and antibody coupling efficiency as illustrated in Table 1. The liposomes were comparable in size and liposomes conjugated with immunoglobulins had similar protein coupling efficiency. The *α*-hEGFR-ILs had a mean diameter of 95.2 ± 3 nm, whereas liposomes conjugated with nonimmune human immunoglobulins (hIgG-ILs) had a mean diameter of 119 ± 12 nm. The size distribution of all liposomes had a polydispersity index <0.2, indicative of a homogenous size distribution. The charge measured of all liposome preparation was slightly negative (Table 1).

### 3.3. *In Vitro* Liposomal Targeting in U87 mg and U251 mg Cell Lines

**Figure 2:**
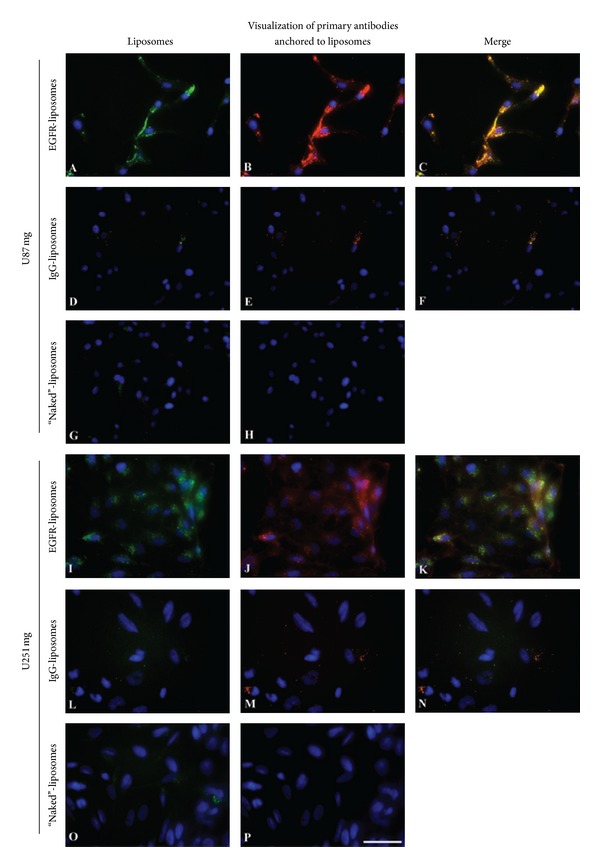
Enhanced uptake of DiO-labeled *α*-hEGFR-IL's in U87 mg and in U251 mg cell lines when compared to hIgG-IL's, or naked liposomes incubated with the cells for 2 hours. (A), (I) DiO-labeled liposomes (green) are only seen in cells incubated with anti-EGFR antibody conjugated liposomes. (C), (K) Verification of co-localization between green DiO-labeled *α*-hEGFR-IL's ((B), (J) red color) in merged overlays seen as yellow color. Cellular nuclei are visualized with DAPI. Scale bar = 50 *μ*m.

Cellular binding and uptake of the three different DiO-labeled liposomes were evaluated by fluorescent microscopy and flow cytometry in the two cell lines. Liposomes were added at a concentration of 75 nmol/10^5^ cells and incubated for two hours at 37°C.The targeting efficiency of *α*-hEGFR-ILs was considerably higher in both U87 mg and U251 mg cell lines (Figures 2(A) and 2(I)) compared to that of hIgG-ILs or naked liposomes (Figures 2(D), 2(G), 2(L), and 2(O)). To verify that the liposomes retrained their conjugation with the anti-EGFR antibodies after internalization, the liposomes were also labeled with a secondary antibody detecting human immunoglobulins (Figures 2(B), 2(E), 2(H), 2(J), 2(M), and 2(P)), which showed that the anti-EGFR antibodies co-localized with the fluorescence emitted by that of DiO of the liposome (Figures 2(C) and 2(K)). Nonspecific binding of the secondary antibody was not observed in the samples exposed to the naked liposomes, which indeed verify the conjugation efficiency of the antibodies to the liposomes.

**Figure 3:**
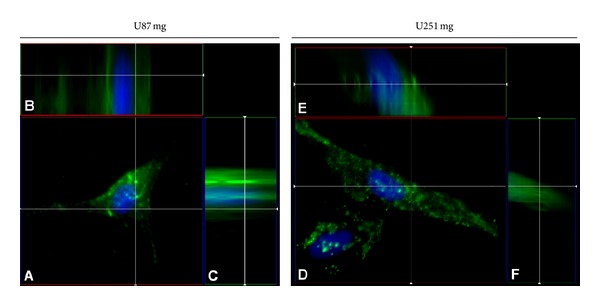
Cellular internalization of DiO-labeled *α*-hEGFR-IL's in U87 mg ((A)–(C)) and U251 mg cell lines ((D)–(F)) as detected by 3D deconvolution of a 25 iteration Z-stack. Note the intracellular localization of DiO-labeled liposomes. Cellular nuclei are visualized by DAPI (blue).

To assess the putative cytoplasmic accumulation through receptor-mediated endocytosis of *α*-hEGFR-ILs in the two cell lines, a Z-stack was obtained from the fluorescent images (Figure 3). A 3D deconvolution analysis was carried out to neutralize scattered light emitted from different focal planes in the Z-stack. The 3D deconvolution confirmed that *α*-hEGFR-ILs were internalized by the cells and accumulated at high density within the cell cytoplasm without labeling the nucleus in both U87 mg (Figures 3(A)–3(C)) and U251 mg cells (Figures 3(D)–3(F)).

### 3.4. Flow Cytometric Analysis of Liposomal Binding and Cellular Uptake

**Figure 4:**
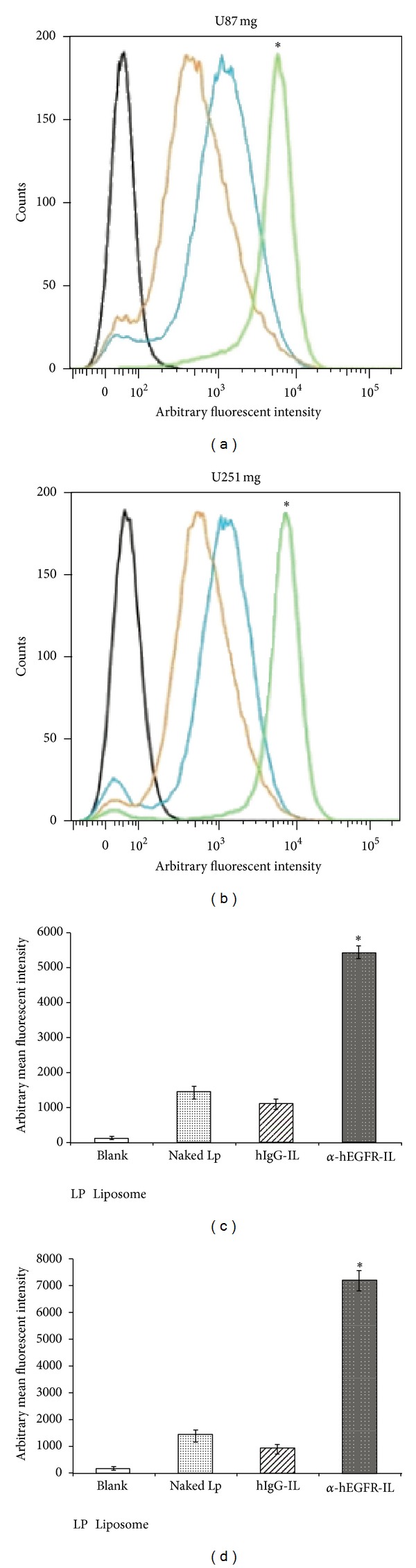
FACS analysis showing enhanced cellular binding of *α*-hEGFR-IL's in U87 mg (a) and U251 mg (b) cell lines. The targeting efficiency of the *α*-hEGFR-IL's (green histograms) was evaluated by comparing mean fluorescence intensities (MFI) with hIgG-IL's (orange histograms), or naked liposome (blue histograms) and cells not exposed to liposomes (black histograms). (c), (d) Comparison of the liposomal MFI of U87 mg (c) and U251 mg (d). *P < 0.05, Lp, liposome.

The findings from the FACS analyses revealed results consistent with those observed in the fluorescent microscopy analyses showing a significant uptake *α*-hEGFR-ILs (Figure 4). Hence, the binding and uptake of *α*-hEGFR-ILs were significantly higher as compared with those of nonimmune immunoglobulin conjugated liposomes or naked liposomes in both the U87 mg and U251 mg cell lines (*P* < 0.05).

### 3.5. Characterization of the U87 mg Tumor-Induced Intracranial Xenograft

**Figure 5:**
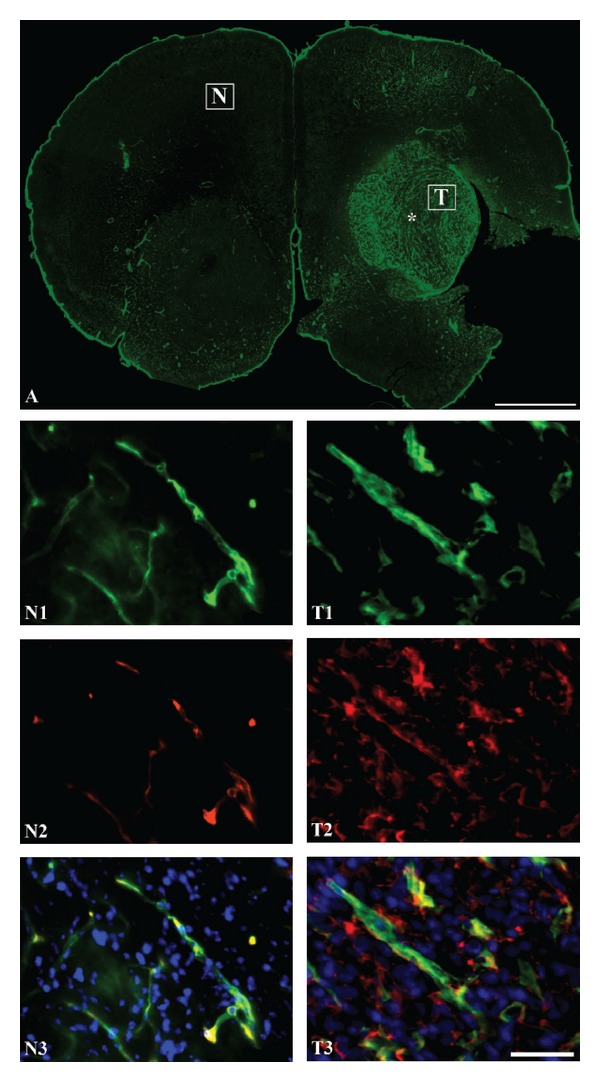
(A), Immunohistochemical characterization of the U87 mg induced intracranial tumor with regard to vascular density as detected by laminin-immunohistochemistry (A, T1) and permeability of albumin (T2). The density of capillaries is clearly higher within the tumor (T) marked with an asterisk compared to that of an area unaffected by tumor formation (N). (N2), (T2) Albumin-immunoreactivity (red) is present within the tumor indicative of a permeable vasculature, whereas albumin is seen occasionally only within the lumen of the brain capillaries. (N3), (T3) Overlays showing that endogenous albumin is present in the interstitium of mouse brain tumor tissue (T3), which contrasts that of the normal brain (N3). Cellular nuclei are visualized DAPI. Scale bar = 50 *μ*m (N1–T3), 1 mm (A).

The tumor formation was examined macroscopically and verified by fluorescence microscopy in cryosections of the mouse brain injected with U87 mg cells (Figure 5). To access the vasculature, an immunohistochemical profile was performed to detect laminin of the basal membrane and endogenous plasma albumin as a marker of permeability (Figure 5). The vasculature between the normal brain and the tumor differed significantly. Hence, the vessels of the tumor were denser, larger in diameter, and overall very irregular compared with those of normal brain vessels (compare Figure 5(N1) with Figure 5(T1)). Endogenous mouse albumin was observed to accumulate extensively in the tumor interstitium indicative of higher permeability to macromolecules whereas, in the normal mouse brain, albumin was only confined to the vessels without appearance in the brain parenchyma indicative of an intact blood-brain barrier (compare Figures 5(N2) and 5(T2)).

### 3.6. Accumulation of Liposomes *In Vivo*

**Figure 6:**
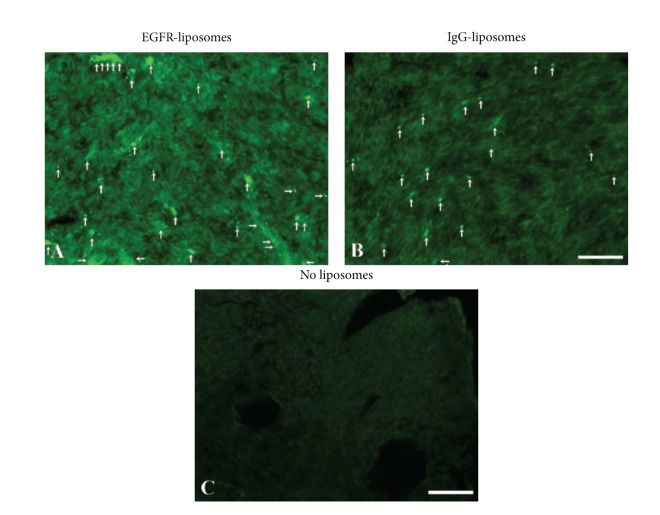
Representative sections containing the U87 mg xenograft tumor showing accumulation of green fluorescent *α*-hEGFR-IL's (A). In comparison, hIgG-IL's accumulate to a lower degree within the tumor (B), but the fluorescence is clearly higher than that of background fluorescence obtained from U87 mg xenograft tumor not injected with liposomes (C). Arrows illustrate accumulation of liposomes within the tumor. Scale bar = 50 *μ*m.

At 4 hr postinjection, the *α*-hEGFR-ILs clearly appeared more prominent within the tumor compared to those of the hIgG-ILs (Figure 6). The presence of *α*-hEGFR-ILs and hIgG-ILs was higher in the periphery of the tumor containing a somewhat higher density of vasculature (Figure 4(a)), whereas in regions with a lower density of vessels, mainly in the center of the tumor, the accumulation of liposomes was drastically decreased (not shown). 

**Figure 7:**
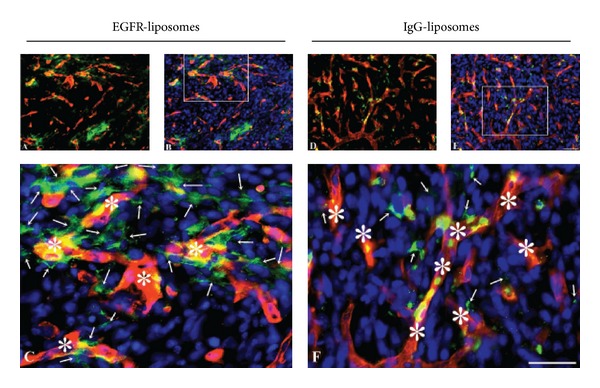
Distribution of green DiO-containing *α*-hEGFR-IL's (A–C) and hIgG-IL's (D–F) in the U87 mg intracranial tumor xenograft co-detected with laminin using a red fluorescent antibody to detect capillaries (asterisks). To enhance the visualization of the green fluorescence emitting DiO-containing liposomes, the sections were additionally incubated with Alexa Fluor 488-conjugated anti-human immunoglobulins. (C), (F) When examined at large magnification (marked areas in (B) and (E)), it is evident that the presence of *α*-hEGFR-IL's is higher within U87 mg cells compared with that of hIgG-IL's (compare 7(C) with 7(F)). Furthermore, it is evident that the green liposomes are present in cells non-labeled in red (arrows). Cellular nuclei identified with DAPI (blue). Scale bar = 50 *μ*m.

To overcome the problem with a relatively weaker fluorescent signal emitted from the green fluorescent emitting DiO-containing liposomes in the tissue sections, the immunoglobulins conjugated to the surface of the liposomes were labeled with additional green fluorescence using an Alexa Fluor 488- conjugated secondary antibody. This allowed for an improved analysis of the section, which revealed that the liposomes indeed localized to the U87 mg cancer cells (Figure 7). The cellular binding to the U87 mg cells was detectable as green fluorescence in the cytoplasma of these cells (Figures 7(A)–7(C)). In sections from mice injected with liposomes conjugated with hIgG-IL's, the liposomes accumulated to a lower degree inside the cancer cells than in cells of sections from mice injected with *α*-hEGFR-ILs (compare Figures 7(C) and 7(F)).

To quantify the appearance of liposomes within the tumor, the mean grayscale intensities (GSI) in brain tumor sections exposed to either *α*-hEGFR-ILs or hIgG-ILs were compared to sections containing a brain tumor from a mouse that was not injected with liposomes (Table 2). The mean GSI in sections of tumors containing *α*-hEGFR-ILs was 28.8 whereas sections of tumors hIgG-ILs were 17.2 corresponding to 1.67-fold higher accumulation of *α*-hEGFR-ILs in the intracranial U87 mg xenograft model (Table 2). This corresponded to that of the mean GSI of sections from tumors containing *α*-hEGFR-ILs, which were observed to be 3.39-fold higher relative to sections containing brain tumor from a mouse that was not injected with liposomes. This was comparable with a 1.95-fold change increase in the mean GSI of tumor sections containing hIgG-ILs (Table 2).

## 4. Discussion

*In vivo* studies reveal that immunoliposomes conjugated with different ligands to target specific tumor antigens, for example, VCAM-1 [16], interleukin-13 [17], and EGFR [18], may be of important clinical significance as a novel treatment for cancer. Immunoliposomes directed against multiple tumor antigens, for example, EGFR and VCAM-1 could, increase the therapeutic efficacy and, hereby, immunoliposomal therapy could become clinically significant as a novel treatment for cancer.

EGFR overexpression by cancer cells is indicative of this ligand-receptor complex role in the pathogenesis of GBM [3, 4]. Upon ligand binding to the receptor, rapid cellular internalization of the receptor-ligand complex will occur [9], which makes the EGFR an interesting candidate for targeted therapy also in GBM. The expression of EGFR in experimental GBM and its antibody-mediated targetability both *in vivo* and *in vitro* were the focus in the present study. Consistent with the findings of the present study, the EGFR expression in the two GBM-based cell lines U87 mg and U251 mg is prominent both *in vitro* [19, 20] and *in vivo* in experimental xenograft models [21, 22]. The cellular binding and uptake of *α*-hEGFR-IL were evaluated in the U87 mg and U251 mg cells and compared with hIgG-IL and naked liposomes *in vitro*. These studies were carried out to assess the potential of targeted therapy for GBM using *α*-hEGFR-IL. *α*-hEGFR-IL demonstrated significant binding in both cell lines versus control liposomes (hIgG-IL and naked liposomes), indicating substantial specificity of *α*-hEGFR-IL. The liposomes used in this study had a mean size distribution of 95 nm (*α*-hEGFR-IL), 119 nm (hIgG-IL), and 83 nm (naked liposomes) and are comparable with other studies using U87 mg as a tumor model to study liposome transport in an experimental model of GBM (e.g., [23]).

Rapid internalization of the receptor-ligand will occur upon binding to the EGFR [1], which makes the EGFR an interesting candidate for targeting therapies in GBM. The monoclonal antibody Cetuximab without liposome conjugation is currently in clinical trials for GBM immunotherapy, and it is approved for treatment of colon cancer [24]. Liposomal targeting of cancer cells to this date has only been investigated in preclinical animal studies. One of the primary aims was to test a model for *in vitro* and *in vivo* anti-EGFR liposomes targeting using U87 mg and U251 mg cell lines. The liposomes were PEGylated at the surface of the liposomes, which has been well documented to increase the half-life of the liposomes *in vivo* [23, 24]. The zeta potential of liposomes also has a significant effect on the targeting efficiency, as cationic liposomes are more readily cleared from the blood stream by the liver and have higher affinity to blood vessels. Anionic liposomes are often rejected from further by the blood vessels; therefore, neutral charged liposomes are optimal for drug delivery. Both the EGFR-IL and hIgG-IL used in this study were slightly anionic, but not to an extent that would affect the efficiency of drug delivery to the tumor. Thus, the properties of these liposomes were in accordance with other studies applying liposomes for targeting purpose *in vitro* and *in vivo* [23].

When comparing brain tumor cryosections gray scale intensities for EGFR-IL and hIgG-IL, an increase of 3.39 fold versus 1,95-fold change could be observed above the background fluorescence of the tumor tissue. This clearly demonstrated a preferential accumulation of the EGFR-immunoliposomes within the tumor tissue. Both *α*-hEGFR-IL and hIgG-IL occurred inside the tumor blood vessels. Additionally, both the *α*-hEGFR-IL and the hIgG-IL were accumulating in the tumor interstitium, which is likely due to the EPR-effect where liposomes regardless of conjugation will accumulate gradually over time [14, 17, 24]. The liposomes trapped in the tumor interstitium by this EPR-effect have substantially increased cellular binding when liposomes were conjugated with anti-EGFR antibody.

The EGFR labeling *in vivo* using the Cetuximab antibody demonstrated good affinity for the EGFR-expressing U87 mg cells, which supports prior findings demonstrating a preferential accumulation of *α*-hEGFR-IL and even increased tumor growth inhibition compared with naked liposomes in a subcutaneous xenograft model [18]. As part of the current study, it was observed that blood vessels of the U87 mg xenograft tumor grown in a cranial microenvironment have significantly smaller pore cutoff-size than xenograft tumors grown in a subcutaneous microenvironment (unpublished observation), which is explainable by the influence of cells denoting the neurovascular unit [14].

Enhanced permeability of macromolecules seen in solid tumor is well documented [14, 25, 26]. Here, we show that the EPR effect is present in the U87 mg intracranial xenograft model by means of the accumulation and retention of endogenous mouse albumin and liposomes in the tumor interstitium. Albumin accumulation in the U87 mg intracranial tumors in mice also exhibited a significantly higher accumulation when examined in another human GBM cell line (HGL21) used for intracranial xenograft formation [25]. The tumor blood vessels generated when using U87 mg for intracranial xenografts are small (100 nm) compared with many other cell lines (500 nm) [14]. In our study, U87 mg intracranial xenografts also displayed high vascularization, but the center of the tumor was less vascularized compared with the tumor periphery. This was not unexpected, since necrosis of tumors is often seen due to their rapid growth.

## 5. Conclusions

In summary, the results obtained from the current study demonstrate that *α*-hEGFR-IL has superior cellular binding compared with control liposomes *in vitro*. Furthermore, the results show that *α*-hEGFR-IL achieved favorable cellular tumor binding in an intracranial xenograft model. This endorses *α*-hEGFR-IL as a good candidate for targeted drug delivery purposes in targeted therapeutic approaches for treatment for GBM in future clinical studies.

## Figures and Tables

**Table 1:** Characterization of liposomes with respect to particle size, polydispersity, charge, and protein coupling yield.

	Particle size (nm)	Polydispersity	Zeta potential mV	*µ*g protein/*µ*mol PL
*α*−hEGFR-IL	95.2 ± 3	0.181 ± 0.045	−1.99 ± 0.067	97.6
hIgG-IL	119 ± 12	0.199 ± 0.091	−0.042 ± 0.042	135.1
Naked liposomes	84.24 ± 5	0.079 ± 0.064	−0.06 ± 0.023	N/A

EGFR-IL: liposomes coated with antiepidermal growth factor receptor (EGFR) antibody; hIgG-IL: liposomes coated with non-immune IgG; naked liposomes: liposomes without antibody conjugated to the surface; PL: phospholipids.

**Table 2:** Arbitrary fluorescent intensity of EGFR-IL and hIgG-IL 4 hours after injection.

	Mean GSI	S.D.	Fold change relative to blank mean GSI	Fold change relative to hIgG-IL
*α*-hEGFR-IL	28.8	8.58	3.39	1.67
hIgG-IL	17.2	6.42	1.95	1
Blank	8.5	N/A	1	N/A

NA: not available.

## Abbreviations

*α*-hEGFR: Anti-human epidermal growth factor receptor antibodies
CNS: Central nervous system
DAPI: 4′,6-diamidino-2-phenylindole
EGFR: Epidermal growth factor receptor
EPR: Enhanced penetration and retention
GBM: Glioblastoma multiforme
IL: Immunoliposome.
